# A rare variant of anomalous left coronary artery from pulmonary artery syndrome

**DOI:** 10.1093/ehjcr/ytaf206

**Published:** 2025-04-26

**Authors:** Hui Wang, Xue-Chen Qiao, Zhen-Gang Zhao, Yuan Feng

**Affiliations:** Department of Cardiology, West China Hospital, Sichuan University, 37 Guoxue Road, 610041 Chengdu, China; Department of Cardiology, West China Hospital, Sichuan University, 37 Guoxue Road, 610041 Chengdu, China; Department of Cardiology, West China Hospital, Sichuan University, 37 Guoxue Road, 610041 Chengdu, China; Department of Cardiology, West China Hospital, Sichuan University, 37 Guoxue Road, 610041 Chengdu, China

## Case description

**Figure 1:**
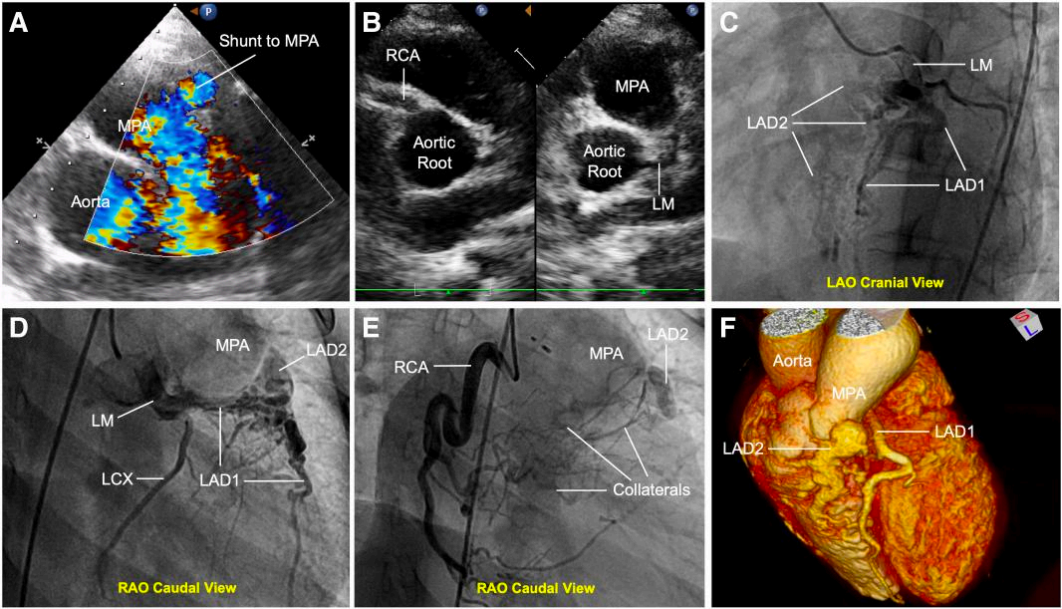
Dual left anterior descending (LAD) variant of anomalous left coronary artery from the pulmonary artery (ALCAPA). (*A*) Transthoracic echocardiography [main pulmonary artery (MPA) long-axis view] showing diastolic shunt (arrow) to the main pulmonary artery. (*B*) Aortic short-axis view demonstrating normal origins of the left main (LM) and right coronary arteries (RCA). (*C* and *D*) Coronary angiography (left and right anterior oblique cranial views) revealing LAD1 and left circumflex artery from left main and anomalous LAD2 from main pulmonary artery. (*E*) Computed tomography angiogram illustrating retrograde filling of LAD2 (arrowhead) and main pulmonary artery opacification via collaterals. (*F*) 3D computed tomography reconstruction confirming dual left anterior descending anatomy.

A 15-year-old female presented with exertional dyspnoea and chest tightness. She had undergone patent ductus arteriosus closure and balloon pulmonary valvuloplasty 9 years prior. Physical examination revealed cyanosis (peripheral oxygen saturation: 89%). Transthoracic echocardiography identified two atrial septal defects (ASD; 9 and 6 mm) with bidirectional shunt, mild pulmonary stenosis, and moderate regurgitation. A 3-mm diastolic shunt to the main pulmonary artery (MPA) was noted (*Figure 1A*). Although the LM and right coronary artery (RCA) appeared normally positioned (*Figure 1B*), coronary angiography revealed a dual left anterior descending (LAD) variant of anomalous left coronary artery from pulmonary artery: LAD1 originated from the LM, while LAD2 anomalously arose from the MPA (*Figure 1C* and *D*). Retrograde filling of LAD2 and MPA opacification via collaterals from LAD1 and RCA were evident (*Figure 1E*). Severe ostial stenosis of LAD2 limited coronary-to-pulmonary shunting. Coronary computed tomography angiography confirmed the anatomy (*Figure 1F*). The patient underwent LAD2 reimplantation without ASD closure (planned for subsequent surgery) and was discharged uneventfully.

In this case, the key factors contributing to delayed diagnosis included: Severe ostial stenosis of LAD2, reducing coronary steal and masking ischaemia until adolescence. Prior severe pulmonary stenosis, which obscured echocardiographic detection of abnormal flow before valvuloplasty. Preserved LM and RCA origins, misleading initial assessment despite dual LAD anatomy. Inadequate post-intervention follow-up, delaying recognition of progressive collateralization.

This variant’s unique antegrade flow via LAD1 combined with collateral-dependent retrograde perfusion explain preserved ventricular function. However, surgical intervention remains critical to eliminate steal and prevent long-term complications.

## Data Availability

The data underlying this article will be shared on reasonable request to the corresponding author.

